# A randomized controlled trial protocol for the introduction of a multidisciplinary individualized nutritional intervention in children with cerebral palsy

**DOI:** 10.1016/j.conctc.2024.101343

**Published:** 2024-08-07

**Authors:** Ruzha Pancheva, Stanka A. Fitneva, Rositsa Chamova, Dimitar Marinov, Albena Toneva, Stanislava Hadzhieva, Rozalina Braykova, Nikoleta Yoncheva, Stefka Tsvetanova, Silviya Nikolova, Natalya Usheva, Koen Huysentruyt, Karina Dimova, Yana Bocheva, Stanislava Pavlova, Petya Hristanova

**Affiliations:** a Department of Hygiene and Epidemiology, Faculty of Public Health, Medical University “Prof. Dr Paraskev Stoyanov”, Varna, Bulgaria; b Research Group NutriLect, Department of Neuroscience, Research Institute, Medical University “Prof. Dr. Paraskev Stoyanov”, Varna, Bulgaria; c Department of Psychology, Queen's University, Kingston, Canada; d Karin Dom Foundation, Varna, Bulgaria; e Department of Social Medicine and Healthcare Organization, Faculty of Public Health, Medical University “Prof. Dr Paraskev Stoyanov”, Varna, Bulgaria; f Department of Paediatric Gastroenterology, UZ Brussel, Vrije Universiteit Brussel (VUB), Brussels, Belgium; g Department of Clinical Laboratory, Medical University “Prof. Dr. Paraskev Stoyanov”, Varna, Bulgaria; h Department of Speech Therapy and Medical Pedagogics, Faculty of Public Health, Medical University “Prof. Dr Paraskev Stoyanov”, Varna, Bulgaria

**Keywords:** Cerebral palsy, Nutrition, Intervention, Individualized, Multidisciplinary

## Abstract

**Introduction:**

Children with Cerebral Palsy (CP) encounter substantial nutritional challenges that impair their health and quality of life. Despite the importance of nutrition in managing CP and the recognition of physiological, behavioral, and social causes of malnutrition, research on the effectiveness of individualized nutritional interventions developed and supported by multidisciplinary teams is scarce.

**Aim:**

The study will evaluate the impact of an individualized nutritional intervention developed and supported by a multidisciplinary team on the anthropometric outcomes and overall health of children with CP.

**Methods:**

A single-center, randomized controlled trial, conducted at the Medical University of Varna, Bulgaria, will enroll 100 children aged 2–12 years and diagnosed with CP. Participants will be randomly assigned to either an intervention group, receiving comprehensive structured dietary assessment and individualized nutrition plan developed by a multidisciplinary team of experts, or to a standard care group. Outcomes assessed will focus on anthropometric measures of nutritional status, but also include health outcomes, child development and clinical assessments, and quality of life indicators.

**Ethics:**

Ethical approval for this study has been obtained from the Medical Ethics Committee at the Medical University of Varna (Protocol No. 134 dated 20.07.2023).

**Conclusion:**

This study will assess the benefits of a multidisciplinary, individualized nutritional intervention for children with CP. The findings will have implications for clinical guidelines and interventions aiming to improve their care and quality of life.

## Abbreviations

BMIAZ: Body Mass Index for Age Z-score
CP: Cerebral Palsy
DP-3: Developmental Profile-3
EDACS: Eating and Drinking Ability Classification System
ESPGHAN: European Society for Paediatric Gastroenterology, Hepatology, and Nutrition
FFQ: Food Frequency Questionnaire
GMFCS: Gross Motor Function Classification System
HAZ: Height for Age Z-score
ITT: Intention-To-Treat
MUACAZ: Mid-Upper Arm Circumference for Age Z-score
PP: Per-Protocol
PEDI-EAT: Pediatric Evaluation of Disability Inventory - Eating Module
QoL: Quality of Life
RCT: Randomized Controlled Trial
SFTAZ: Skinfold Thickness for Age Z-score
SPIRIT: Standard Protocol Items: Recommendations for Interventional Trials
WAZ: Weight for Age Z-score
WHO: World Health Organization

## 1 Introduction

Cerebral Palsy (CP) encompasses a group of permanent movement and posture disorders, leading to activity limitations due to non-progressive disturbances in the developing fetal or infant brain (Rosenbaum et al., 2007). As the most prevalent physical disability in childhood, CP affects approximately 1.6–3.4 per 1000 live births globally [1].

Children with CP encounter numerous health challenges, including malnutrition, which aggravates their physical limitations and negatively impacts their own quality of life (QoL) and that of their families [2]. Malnutrition in CP results from feeding difficulties, inadequate dietary intake, elevated energy expenditure, and malabsorption, highlighting the need for tailored nutritional interventions [3].

While nutritional interventions are pivotal for enhancing nutrient intake, growth, functional abilities, and thus QoL, empirical evidence supporting their effectiveness in CP is sparse. High-quality, randomized controlled trials (RCTs) are imperative to inform clinical practices [4].

Current literature reveals a significant knowledge gap in designing and implementing effective, practical, and family-friendly comprehensive nutritional interventions for children with CP. This RCT aims to bridge this gap by evaluating a nutritional intervention's efficacy in promoting growth and QoL, and in reducing bedridden days among children with CP. The ultimate objective is to establish an evidence-based framework for nutritional interventions within clinical care for children with CP, potentially enhancing their health outcomes and QoL.

In many countries, including Bulgaria, nutritional assessment and management are not standardized components of clinical care. This study presents an ethical opportunity to examine if such practices can effectively improve patient well-being, beyond existing clinical and family-led care, particularly in contexts like Bulgaria. Amidst the high costs of medical care—in terms of money, time, and emotional toll—this research advocates for evidence-based healthcare practices and standards.

## 2 Objectives

### 2.1 Primary objective

To evaluate the effect of an individualized nutritional intervention based on multidisciplinary assessment on the nutritional status of children with CP over a six-month period.

### 2.2 Secondary objectives

● ***Healthcare*** utilization outcomes: To assess the nutritional intervention's efficacy in decreasing the number of bedridden days. Optimal nutrition can fortify physical capacities, potentially leading to more active days [5]. Assessing the effect of the intervention at this level could inform health care policy in Bulgaria and abroad.
● Developmental outcomes: To investigate the potential cognitive enhancements associated with the nutritional intervention. Recent research suggests that nutrition has downstream effects on cognitive function [6]. It is unclear, however, whether these effects can be observed as a result of a brief intervention.
● Quality of Life outcomes: To determine the impact of the intervention on enhancing the overall quality of life for children with CP. Improvements in nutrition and physical health can be subsequently reflected in increased well-being and life satisfaction, as captured by established assessment tools [7].

## 3 Design and methods

### 3.1 Study setting

The study will be a prospective, single-center, RCT conducted at the Medical University of Varna. This protocol adheres to the Standard Protocol Items: Recommendations for Interventional Trials (SPIRIT) 2013 [8] statement to prevent study design-related bias.

### 3.2 Participants and recruitment

The target population is 2- to 12-year-old children diagnosed with CP. Participant recruitment will adhere to clearly defined inclusion and exclusion criteria (see Eligibility).

The research team will collaborate with Karin Dom Foundation, a national leader in services for children with disabilities, residential centers, and other relevant organizations to identify potential participants. After an initial phone screening, a psychologist???? will conduct an intake interview with guardians to assess child eligibility. The families of eligible children will be thoroughly briefed on the RCT's aims, benefits, and risks. Written consent from parents or guardians will be required for participation. All personal information will be kept confidential and the data will be anonymized for analysis.

### 3.3 Eligibility

#### 3.3.1 Inclusion criteria

Children aged between 2 and 12 years who have been diagnosed with cerebral palsy by a qualified neurologist will be eligible for the study. The diagnosis of CP can refer to spastic, dyskinetic, ataxic, or mixed CP. Only children of families who intend to continue to reside in the catchment area for the duration of the study will be included.

#### 3.3.2 Exclusion criteria

Children will be excluded for the following reasons: 1) acute medical conditions or severe infections in the 10 days prior to the family being contacted; 2) genetic syndromes affecting nutritional status like Silver Russel or Down syndrome; 3) participation in dietary interventions within the last three months; and 4) guardians do not fully understand the study's terms or don't speak Bulgarian.

### 3.4 Study measurements and outcomes

This trial has comprehensive baseline and final measurements of all trial outcomes (Table 1). Additionally, at baseline we will collect demographic details about the child (age, gender, ethnicity, and socio-economic status of their immediate family), their medical history (focusing on CP diagnosis, the severity of neurological impairments, and any other prevalent medical conditions including comorbid disorders) and their primary caregiver (relationship to the child, educational background, and involvement in the child's daily care).

**Table 1:** Overview of instruments and timepoints.

Questionnaire/Activity	Study start	During study					
	Visit 1 (0 month)	Follow up 1 month (±5 days)	Follow up 2 months (±5 days)	Follow up 3 months (±5 days)	Follow up 4 months (±5 days)	Follow up 5 months (±5 days)	Visit 2 (6 months ± 15 days)
Information for parents	✓						
Signed Informed consent	✓						
Demographic questionnaire	✓						
Collected Health record data	✓						✓
Psychodiagnostic test DP-3	✓						✓
Nutrition questionnaire by Speech therapist	✓						✓
EDACS or mini EDACS	✓						✓
Pedi-EAT_	✓						✓
3-day Food diary	✓						✓
Nutrition Questionnaire about feeding problems and food preferences	✓						✓
Quality of life questionnaire	✓						✓
Anthropometry	✓						✓
Biochemical tests	✓						✓
Follow up interview after Visit 1 (e-mail)				✓			
Follow up interview after Visit 1 (GSM)		✓	✓		✓	✓	

Importantly, child assessments will be conducted by a multidisciplinary team consisting of nutritionists, speech therapists, psychologists, pediatric gastroenterologists. A nutritionist will use the findings from the assessment and collaborate with the team in developing the individualized nutritional plans for children in the intervention arm (see below).

During intake, further assessment of the inclusion and exclusion criteria will occur, alongside evaluation for the presence of comorbid disorders. Family and Child Characteristics will be collected, including:

1 Demographic Details: Age, gender, ethnicity, and socio-economic status of the child and their immediate family
2 Medical Profile**:** A detailed recording of the child's medical history, focusing on CP diagnosis, the severity of neurological impairments, and any other prevalent medical conditions including comorbid disorders.
3 Acute medical conditions Profile: Additional data on the number type and frequency of acute medical issues in the last 6 months will be collected
4 Caregiver's Profile: Information on the primary caregiver's relationship to the child, their educational background, and their involvement in the child's daily care.
5 Developmental assessment will be based on the Developmental Profile-3 test (DP-3) ([9], which evaluates development from birth to age 12 across five functional domains: Physical Development, Adaptive Behavior, Social-Emotional Development, Cognitive Development, and Communication. The DP-3 is conducted in the form of an interview with the child's parents or caregivers, and the instrument is administered by certified psychologists. The developmental assessment scale DP-3 is standardized for Bulgaria, and the obtained quantitative results allow for a comparative analysis with the results of a normative sample by age and gender. Additionally, it enables reporting differences in each of the functional areas of development before and after the application of a given intervention.
6 Quality of Life and Needs Assessment:
  ● Needs Assessment**:** Structured interviews and surveys to explore the effects of the child's neurological challenges on family dynamics and support systems.
  ● Quality of Life Measure: Employing established and validated tools, such as the Beach Center Family Quality of Life Scale, this assessment method systematically gauges families' perceptions across critical dimensions including Family Interaction, Parenting, Emotional Well-being, Physical/Material Well-being, and Disability-Related Support.
7 Dysphagia Evaluation:
  ● Eating Abilities Analysis: Conducted by a speech therapist using the Pediatric Evaluation of Disability Inventory - Eating Module (PEDI-EAT) to assess speech, oral motor function, communication skills, and feeding and swallowing abilities.
  ● Direct Feeding Assessment: In-depth evaluation through clinical examinations, caregiver interviews, and mealtime observations using the Eating and Drinking Ability Classification System (EDACS) and mini-EDACS.
  ● Direct Swallowing Examination**:** Clinical evaluations and oral motor assessments to ensure safety during eating and drinking.
8 Nutritional Status Assessment: Conducted by a nutritionist, including measurements of height, weight, skinfold thickness, and mid-upper arm circumference, with results plotted against WHO standards and CP-specific growth charts. Dietary intake will be assessed using a 3-day food diary, a Food Frequency Questionnaire (FFQ), and evaluations of food and texture preferences, as well as gastrointestinal issues such as constipation, dysphagia, and gastroesophageal reflux.

### 3.5 Randomization

**Fig. 1:**
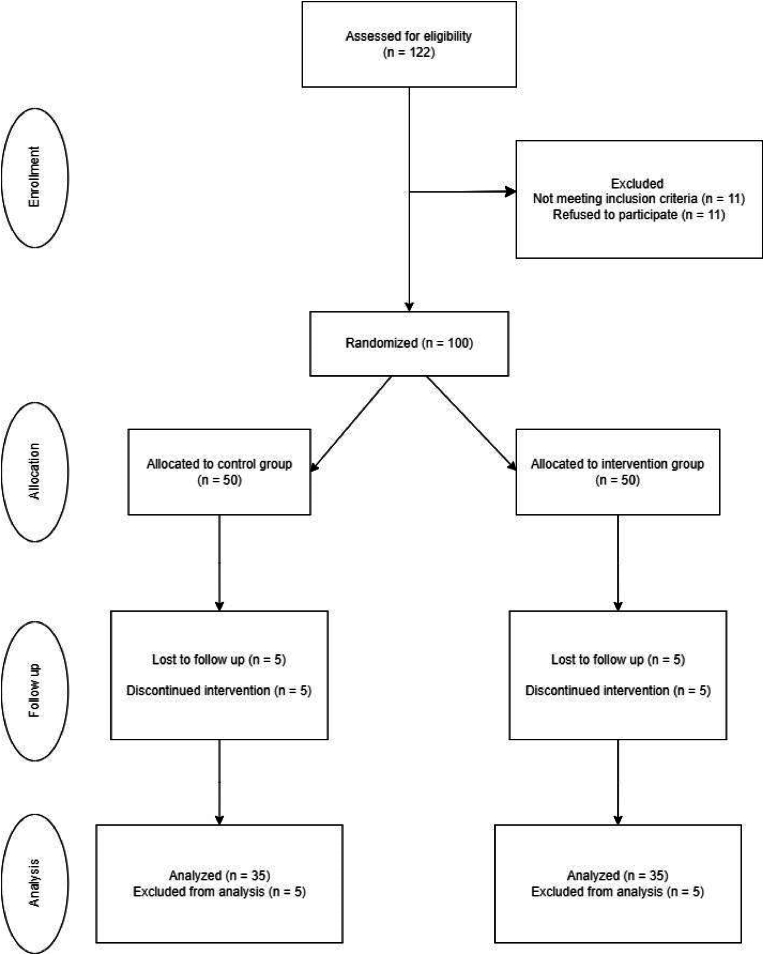
Flowchart of study process.

We aim to enroll 100 children in the study. After successful recruitment, participants will be randomized in a 1:1 ratio into one of two groups: the intervention group, which will receive a comprehensive nutritional intervention, and the control group, which will receive standard care (Fig. 1).

The randomization process will employ a computer-generated sequence, with stratification based on gender and Gross Motor Function Classification System (GMFCS) levels. For the latter, participants will be divided into two groups: those classified on GMFCS levels 1 to 3 and those classified on GMFCS levels 4 and 5 Baranek et al., [10]. Stratification will ensure a balanced representation of motor impairment variation across the intervention and control group.

### 3.6 Intervention

*Nutritional Intervention Arm* The baseline comprehensive nutritional assessment will lay the groundwork for a tailored nutrition plan informed by the assessment outcomes and guided by the national nutrition recommendations for children in Bulgaria [11], registered dietitians will develop personalized dietary recommendations for each child. These recommendations will be designed to address the unique dietary needs and challenges of each participant, taking into careful consideration their individual food preferences, sensitivities, and essential nutritional requirements. Adjustments in diet may include modifications to the texture, taste, and presentation of food, aiming to enhance food acceptance and meet nutritional goals. In certain cases, the introduction of dietary supplements, ranging from enteral formulas to fortified foods or more comprehensive measures like enteral nutrition, is recommended in accordance with ESPGHAN guidelines [12].

The support and monitoring of this intervention are carried out by qualified nutritionists or dieticians. This involves a structured follow-up mechanism that includes monthly phone check-ins and a comprehensive email consultation at the end of the third month. These interactions aim to address any nutrition-related concerns, assess adherence to the dietary recommendations, and make necessary adjustments. The adherence to the nutritional plan is further evaluated during a 6-month visit, primarily through the improvement in dietary intake as documented in a three-day food diary.

### 3.7 Standard care comparison

Participants assigned to the *Standard Care Arm* receive the conventional care typically provided to children with neurological impairments in their respective daycare or residential centers. This standard care encompasses a range of services, including regular health check-ups, therapeutic services, and educational support, aimed at addressing the general needs of children with CP.

### 3.8 Outcomes

#### 3.8.1 Evaluating nutritional progress

The study will assess the nutritional status of participants using key physical measurements, including Body Mass Index for Age Z-score (BMIAZ) when it is possible to obtain a reliable height or length measurement, Weight for Age Z-score (WAZ), Height for Age Z-score (HAZ) or Knee height for Age Z-score (if feasible), Triceps and subscapular Skinfold Thickness for Age Z-score (SFTAZ), and Mid-Upper Arm Circumference for Age Z-score (MUACAZ). Z-scores will be calculated based on WHO growth charts or CDC-charts for skin folds. Weight will be compared with CP-specific growth charts. The transformation in nutritional status is further delineated by alterations in energy intake, captured through detailed analysis of a prospective 3-day food diary. Complementing these measurements, biochemical assessments provide insights into the iron status (ferritin and Hb values), 25-OH-vitamin D levels, and vitamin B12 concentrations, offering a comprehensive view of the nutritional intervention's impact.

#### 3.8.2 Health outcome metrics

Within a six-month framework, the study assesses health outcomes through several lenses:

● The number and duration of days a child remains bedridden, offering insights into the intervention's impact on physical health.
● The frequency of emergency medical visits and incidents of infections, including related hospitalizations, which serve as indicators of health stability and resilience.
● Observations on days when the child's usual activities are hindered due to health issues, as well as instances where the child's health status influences family activities, revealing the broader implications of improved nutritional care.

Signs of malnutrition in CP were widespread, showing a direct link between inadequate nutrition and higher demands on healthcare services, including more frequent hospital stays and doctor appointments. This nutritional deficit also led to reduced engagement in everyday activities for both the child and their parent [13,14].

#### 3.8.3 Child development and behavioral dynamics

The study employs the Developmental Profile 3 (DP-3) to observe changes in scores across developmental and behavioral domains. This tool enables a nuanced understanding of how nutritional interventions may influence cognitive, physical, social, and emotional development milestones.

#### 3.8.4 Quality of life and social well-being

Quality of life changes and the dynamics within the social sphere are meticulously evaluated using validated instruments like the Pediatric Quality of Life Inventory (PedsQ) and the Family Impact Scale. These assessments aim to uncover the physical, emotional, social, and educational shifts experienced by the child and their family, painting a holistic picture of the intervention's effects. Furthermore, structured interviews and surveys delve into the nuances of how the child's neurological challenges and the dietary interventions interplay with family dynamics, support systems, and overall well-being.

### 3.9 Assessment and participant timeline

Progress of the nutritional intervention will be monitored through monthly phone calls and a mail assessment at the third month. Assessments will occur at study entry and six months later, involving demographic and clinical interviews plus questionnaires to evaluate intervention outcomes. This schedule ensures ongoing intervention monitoring and detailed effectiveness data, detailed in Table 1 with validated standard clinical care instruments.

## 4 Planned analyses

**Fig. 2:**
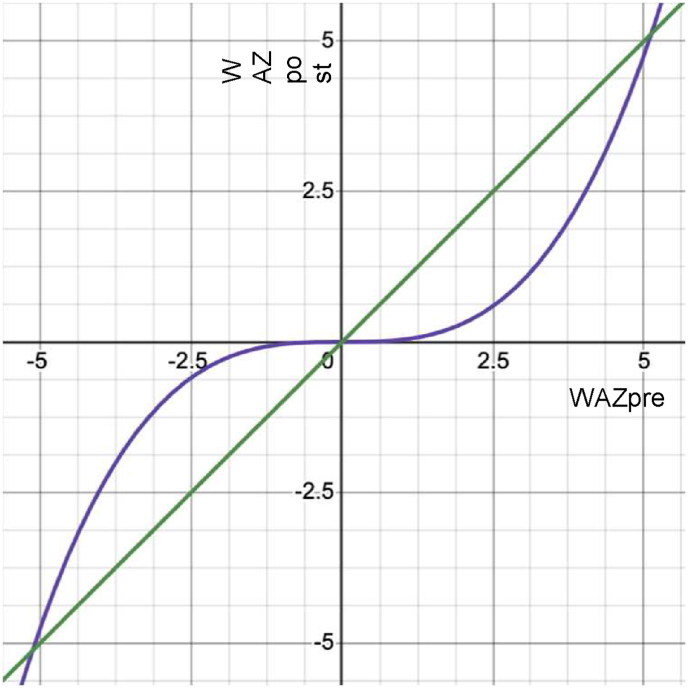
Hypothetical form of the results for control group (green) and intervention group (blue). The figure captures the intended change for underweight and overweight children and the intended no change for children with weight in the normal range. (For interpretation of the references to colour in this figure legend, the reader is referred to the Web version of this article.)

The outcomes of the intervention will be assessed using both Intention-to-Treat (ITT) and Per-Protocol (PP) approaches. In the ITT approach, analyses include all study participants who were randomized to either of the study arms. In the PP approach, the analyses are restricted to participants who adhere to the protocol. We will not perform any imputation for missing data. Typically, the ITT method is favored for trials aiming to demonstrate superiority or non-equivalence (such as our study), PP approach is more suitable for trials assessing equivalence or non-inferiority Ahn et al., [15]. Nonetheless, it is advisable to execute both methods of analysis and compare their outcomes to identify any notable discrepancies. Employing both the ITT and PP strategies offers a fuller understanding of the effects of the treatment and enhances the credibility of the trial findings. In the case of the ITT analysis, we will focus on Weight for age Z-score (WAZ) (based on WHO standards 2006–2007) [16]. As WAZ is a standardized score for an individual given population norms, we anticipate the intervention to produce change at the tails of the distribution (for children outside the normal range) rather than the center (children in the normal range). This suggests that, in the intervention group, the baseline WAZpre variable may have a cubic relationship with the outcome WAZpost variable (Fig. 2).

As Fig. 2 suggests, the expected findings depend on the initial distribution of WAZ. An effect of the intervention may show as a main effect if the participants fall mainly at one end of the WAZ distribution, an interaction between Treatment and Baseline if baseline WAZ extends from one end of the WAZ distribution to 0, or an interaction between the cubed Baseline term and Treatment if baseline WAZ spans the entire WAZ distribution.

Thus, to analyze the change in WAZ, we will first identify the best fitting regression model, starting with [17]. The independent variable Treatment will be coded 0 (control - standard care arm) and 1 (intervention arm). Interaction and cubic terms will be then added, at each step assessing if a more complex model is warranted by the data, based on adjusted R-squared [18] shows the most complex model we expect to assess. We will report the results from the best fitting model.

(1) $${WAZpost\;∼\;b}_{0+b1*Treatment+b2*WAZpre}$$

(2) $$WAZpost\;∼\;b0+b1*Treatment+b2*WAZpre+b3*Treatment*WAZpre+b4*WAZpre^{3}+b5*Treatment*WAZpre^{3}$$

### 4.1 Sample size calculation

Sample size calculations are based on assuming that 1) is the best fitting model. While it is desirable to base sample size on the most complex model used in the analyses, given that 1) it is unclear which model will show best fit to the data, and 2) estimates of sample size for regression models including interactions require understanding of the correlations between the variables, an understanding that we currently do not have, this approach was deemed warranted.

**Fig. 3:**
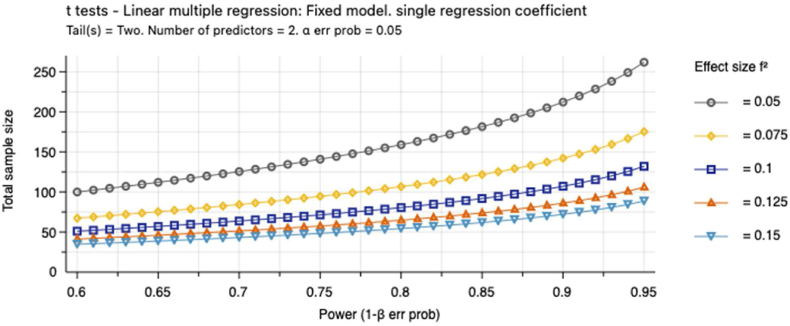
Required sample size expecting small-to-medium effect of the intervention.

Sample size calculations were conducted in G*Power 3.1 1 Fig. 3 shows the sample size calculations assuming that 1) the effect of the intervention will be small to medium, and 2) a two-sided significance test.

We decided to aim for a total sample of 100 participants, which will allow us to detect effects as small as f2 = 0.075 with power of 0.8. Importantly, this sample size also allows us to have reasonable confidence in detecting small-to-medium effects (e.g., f2 = 0.1) assuming a drop out rate as large as 30 % (i.e., final n = 70), which is important for per-protocol analyses.

Secondary analyses will explore nutrition's impact on health and wellbeing in children with CP, focusing on four key areas:

1. Anthropometric Measures: Beyond the primary measure of Weight for Age Z-score (WAZ), we will use BMI, skin fold thickness, and mid-upper arm circumference, using age-standardized z scores, to assess the robustness of our conclusions about the effectiveness of the intervention across anthropometric indicators of nutritional status.
2. Biochemical Indicators: Analyzing Vitamin D and iron levels to identify changes in nutritional deficiencies between intervention and control groups.
3. Healthcare Utilization: We'll assess health indexes such as bedridden days, emergency visits, infections, hospitalizations, and impact on regular activities and family life, comparing improvements between the intervention and control groups.
4. Behavior and Quality of Life: Investigating changes in child behavioral functioning and family quality of life to determine if the intervention group shows greater improvements compared to the control group.

These analyses will deepen our understanding of the nutritional intervention's broader impacts on children with CP and their families.

## 5 Data management

We will implement a strict data management strategy to ensure data integrity, confidentiality, and reliability. Data will be securely collected electronically, with entry by trained personnel. Our protocol adheres to Ref. [19] guidelines and the Declaration of Helsinki, and it has received ethical approval by the Medical Ethics Committee at the Medical University of Varna (Protocol No. 134 dated 20.07.2023). Informed consent will be obtained from parents or guardians after providing them with thorough information about the study. Data privacy is a priority and access will be restricted to authorized personnel. To mitigate bias, the statistical analysis will be blinded. Group identifiers will be replaced with neutral codes by an independent data manager before data analysis. The analyst will conduct the analysis without knowledge of group identities, which will only be revealed after the primary analysis is complete. This blinding process will be thoroughly documented, and an independent statistician will verify the analysis to ensure its integrity. The study is registered on clinicaltrials.gov (NCT06065904).

## 6 Ancillary and post-trial care

Ancillary care will respond promptly to any adverse events, aligning with ethical guidelines to protect participant safety. After the trial, all participants will receive post-trial care to meet ongoing health needs, ensuring the study's benefits continue. Notably, control arm participants will also gain from tailored nutritional support based on the study's findings, ensuring equitable health benefits for all, thus upholding our ethical commitment and enhancing the study's impact.

## 7 Funding

### 7.1 Source of funding

The European Union-NextGenerationEU funds this study through the National Recovery and Resilience Plan of the Republic of Bulgaria, project № BG-RRP-2.004-0009-C02. The funders have no role in study design, data collection and analysis, decision to publish, or preparation of manuscripts.

## 8 Discussion

This study protocol outlines a rigorous approach to evaluating the efficacy of a multidisciplinary individualized nutritional intervention for children with CP. The study would play a pivotal role in shaping future nutritional guidelines and therapeutic strategies in the management of CP.

CP is a complex condition that poses significant nutritional challenges, often leading to malnutrition and poorer health outcomes for these children da Silva et al., [20], [14]. Despite the urgency of the problem, there is a paucity of high-quality research investigating the effects of nutritional interventions in this population [21]. Our study, based on a robust randomized control design, aims to fill this critical research gap and generate much-needed evidence on this important issue.

We hypothesize that our multidisciplinary individualized nutritional intervention will result in improved growth and a decrease in the number of bedridden days, and better overall quality of life for children with CP. These outcomes are based on the understanding that better nutritional status can enhance physical health, improve functional abilities, reduce complications, and promote overall well-being.

The use of an ITT analysis approach in our study will ensure a realistic estimate of the effect of the intervention by accounting for non-compliance and protocol deviations that often occur in real-world settings. This is significant as it enhances the generalizability of our findings.

The success of this study is not only determined by the efficacy of the intervention but also by its acceptability and feasibility for the families involved. It will be essential to take into account the views and experiences of participating families, as their commitment and involvement are integral to the successful implementation of the intervention.

However, it's important to recognize potential limitations. While our study focuses on a single-center, which ensures a uniform approach to intervention implementation and data collection, it might limit the generalizability of our findings. In future, multi-center studies could be valuable for encompassing a wider variety of settings and populations.

Despite the complexities associated with this condition and the potential challenges in implementing the intervention, the study is expected to yield valuable insights that can significantly influence the care and treatment strategies for children with CP.

In a broader context, this study underlines the crucial role of nutritional interventions in managing CP, potentially paving the way for similar research in other complex conditions where nutrition plays a significant role. By harnessing the potential of nutrition, we can enhance health outcomes and quality of life for children with CP and their families, making a tangible difference in their lives.

## 9 Conclusion

In conclusion, this randomized controlled one-center trial aims to evaluate the effect of a multidisciplinary individualized nutritional intervention for children with CP, with a particular focus on nutritional status. It addresses a significant gap in the current literature, providing high-quality evidence to inform clinical practice and guide development in the management of nutritional issues in children with CP. Upon completion, the study findings will be shared with the wider scientific and medical community, contributing to the collective understanding and management of CP, thereby, ultimately contributing to the betterment of pediatric health globally.

## Funding

This study is financed by the European Union-Next Generation EU, through the National Recovery and Resilience Plan of the Republic of Bulgaria, project № BG-RRP-2.004-0009-C02.

## CRediT authorship contribution statement

**Ruzha Pancheva:** Writing – original draft, Methodology, Conceptualization. **Stanka A. Fitneva:** Writing – original draft, Methodology. **Rositsa Chamova:** Writing – review & editing. **Dimitar Marinov:** Writing – review & editing. **Albena Toneva:** Writing – review & editing. **Stanislava Hadzhieva:** Writing – review & editing. **Rozalina Braykova:** Writing – review & editing. **Nikoleta Yoncheva:** Writing – review & editing, Conceptualization. **Stefka Tsvetanova:** Writing – review & editing, Conceptualization. **Silviya Nikolova:** Writing – original draft, Project administration, Methodology, Conceptualization. **Natalya Usheva:** Writing – review & editing, Conceptualization. **Koen Huysentruyt:** Writing – review & editing, Conceptualization. **Karina Dimova:** Writing – review & editing. **Yana Bocheva:** Writing – review & editing, Conceptualization. **Stanislava Pavlova:** Writing – review & editing. **Petya Hristanova:** Writing – review & editing.

## Declaration of competing interest

The authors declare that they have no known competing financial interests or personal relationships that could have appeared to influence the work reported in this paper.
